# Phenomenological dynamics of COVID-19 pandemic: Meta-analysis for adjustment parameters

**DOI:** 10.1063/5.0019742

**Published:** 2020-10-20

**Authors:** Sergio A. Hojman, Felipe A. Asenjo

**Affiliations:** 1 Departamento de Ciencias, Facultad de Artes Liberales, Universidad Adolfo Ibáñez, Santiago 7491169, Chile; 2 Departamento de Física, Facultad de Ciencias, Universidad de Chile, Santiago 7800003, Chile; 3 Centro de Recursos Educativos Avanzados, CREA, Santiago 7500018, Chile; 4 Facultad de Ingeniería y Ciencias, Universidad Adolfo Ibáñez, Santiago 7491169, Chile

## Abstract

We present a phenomenological procedure of dealing with the COVID-19 (coronavirus disease 2019) data provided by government health agencies of 11 different countries. Usually, the exact or approximate solutions of susceptible–infected–recovered (or other) model(s) are obtained fitting the data by adjusting the time-independent parameters that are included in those models. Instead of that, in this work, we introduce dynamical parameters whose time-dependence may be phenomenologically obtained by adequately extrapolating a chosen subset of the daily provided data. This phenomenological approach works extremely well to properly adjust the number of infected (and removed) individuals in time for the countries we consider. Besides, it can handle the sub-epidemic events that some countries may experience. In this way, we obtain the evolution of the pandemic without using any *a priori* model based on differential equations.

Our understanding on the evolution of any pandemic is as good as the model used to fit the data that we can recollect, as well as the quality of the gathered data. The limitations of any model lie in how we mathematically describe our lack of knowledge about the propagation of the virus. The most widely used mathematical models describe such propagation through a set of differential equations with constant parameters. Those encompass the unknown behavior of the propagation of the virus, which depends on every specific society. In this work, rather than construct a mathematical model based in differential equations, we propose that the behavior of the virus in each population is coded in the evolution of such “constant” parameters. It is shown that those parameters evolve over time, and their evolution may be deduced. These new meta-parameters allow us to extract the information of the pandemic day-by-day, in a dynamical way, as the pandemic evolves and the different government policies and society behavior change in time. Thus, we describe the epidemic evolution without solving differential equations.

## I. INTRODUCTION

Pandemic propagation models are usually described by systems of first-order ordinary non-linear coupled differential equations such as is the case for the well-known susceptible–infected–susceptible (SIS) and susceptible–infected–recovered (SIR) models. Numerous previous, as well as very recent, articles have been written to deal with this problem.^1–9^ The dynamical variables in such models are usually denoted by S(t), I(t), and R(t), which are functions of a single time variable t and denote the number of susceptible individuals (which may get infected), the number of infected individuals, and the number of recovered or removed individuals (which sometime after becoming infected are either immune or die), respectively.

In addition to the dynamical variables, the models introduce time-independent parameters (usually denoted by greek letters) that describe the intensity of the coupling between the variables. These parameters clearly depend on the behavior of quarantined people in different countries, which, in turn, depend on the containment policies implemented by different governments in different countries.

It is not difficult to realize, when trying to fit the data informed by health governmental institutions of different countries, that they cannot be fitted by solutions of the model’s equations for time-independent parameters since policies change over time and so does the behavior of the societies. Strictly speaking, the so-called parameters are dynamical variables whose time evolution equations are difficult to hypothesize or construct because their time evolution depends on people's idiosyncrasies and government policies, which are almost impossible to foresee. To overcome this issue, some (or all) of the parameters in those pandemic models can be promoted to time-dependent variables (see, for example, Refs. 10–21). However, the exact time dependence is still unknown in those new variables, and the evolution of the pandemic is still very sensitive to the used model. This has been explicitly shown, for example, in the study of applications of travel restrictions during the spread of coronavirus disease 2019 (COVID-19) pandemic,^22^ concluding that more realistic models must consider the time changing behavior of pattern population to model a large-scale development of diseases.

Because of these difficulties, in this work, we propose an analysis on the study of the daily change of such parameters in order to retrieve the dynamical information of a pandemic. The estimation of the time-dependent behavior of the parameters of epidemiological models has been an active research field in the past.^10–21^ However, our procedure is different from previous ones, as it does not require any set of differential equations as a model. Instead of studying the total dataset that gives origin to the total structure of the pandemic, we focus on the study of time evolution of the parameters that produce such a total structure; i.e., we do a meta-analysis of the dataset system. Therefore, rather than solving a model described by a set of differential equations with solutions that fit the data, a meta-analysis proceeds in the opposite direction, finding the global evolution of the parameters and thus obtaining a model. This procedure allows us to find the global dynamical behavior of data by studying the day-to-day evolution of the adjustment parameters. In this way, we can extract the time-dependent information of the system without solving any differential equation set. Hence, we are able to solve the system in a phenomenological fashion. This proper meta-analysis of the adjustment parameters can provide the kind of information that is needed to have a better understanding of the evolution of the pandemic. The main goal of this work is to show that this procedure gives us global information about the changes in the spread of the disease.

The adjustment parameters in this meta-analysis, hereafter called meta-parameters, are no longer considered as constants, and they can be extracted directly from the data. It is the purpose of this work to delineate a systematic procedure to estimate the dynamics of meta-parameters. We show how these meta-parameters substantially improve the understanding of the global evolution of the pandemic. This is exemplified in the case of data from 11 countries. These are Italy, the United States, Canada, the United Kingdom, Spain, Poland, Austria, Germany, Portugal, New Zealand, and France.

In Sec. II, we briefly describe the SIR model in order to highlight the differences with the current proposed model. Then, in Sec. III, we introduce the phenomenological model for pandemic dynamics and later, in Sec. IV, apply it to 11 countries. We end with a discussion of our proposal.

## II. SIR MODEL

To put our proposal in context, let us consider first the SIR model as an example of pandemic evolution. This model considers three kind of populations, the susceptible S=S(t), the infected I=I(t), and the recovered R=R(t) population, respectively, all of them evolving in time. Besides, the total population N=S+I+R is constant in time. The three variables are related by the differential system $\dot{S}=-\alpha SI$, $\dot{I}=\alpha SI-\beta I$, and $\dot{R}=\beta I$ [where $\dot{A}(t)\equiv dA(t)/dt$]. Here, α and β are constant parameters that contain the relevant information for pandemic evolution. Our lack of knowledge of how the pandemic evolves is hidden in such parameters. Although no explicit exact solution for R=R(t) is known, it is straightforward to show that at second order in an expansion around αR/β, we can obtain the solution for the recovered population as a function of time,^3,23^ given by $R(t)\approx r_{1}\tanh(r_{2}\;t-r_{3})+r_{4}$. Here, $r_{1}=\gamma \beta^{2}/(\alpha^{2}S_{0})$, $r_{2}=\beta \gamma /2$, $r_{3}=\tanh^{-1}\left(\alpha S_{0}/(\beta \gamma )-1/\gamma \right)$, and $r_{4}=\beta /\alpha -\beta^{2}/(\alpha^{2}S_{0})$ are all constants in terms of $\gamma ={\left[{\left(\alpha S_{0}/\beta -1\right)}^{2}+2S_{0}I_{0}\alpha^{2}/\beta^{2}\right]}^{1/2}$, where $S_{0}$ and $I_{0}$ are the initial values of susceptible and infected populations at t=0. It has been assumed that the initial value of the recovered population $R_{0}=0$. It is important to realize that the system is now completely solved, as the infected population can be readily obtained by $I(t)=\dot{R}/\beta \approx (r_{1}\gamma /2){\text{sech}}^{2}(r_{2}\;t-r_{3})$, while the susceptible population is S(t)=N−I(t)−R(t). Those solutions are often used to study the pandemic evolution in an approximated manner. However, they fail to describe correctly its dynamics when social conditions change or different governmental decisions are taken along the progress of the pandemic.

In Secs. III–V, we show how better fitting results can be achieved by the procedure of using a hyperbolic tangent function to fit the data of the recovered population during the pandemic, as a starting point for the procedure using meta-parameters. The evolution of these meta-parameters is obtained by analyzing day-by-day the same data that they adjust. This procedure gives a precise figure of the increment of infected individuals. Therefore, the meta-analysis produces a better fitting of the estimation of the temporal behavior of the infected population, thus solving the pandemic dynamics in a phenomenological way. The final solution obtained from the data fitting procedure will not be a solution of the SIR model, neither of any other simple model described by first-order differential equations.

## III. PHENOMENOLOGICAL TREATMENT FOR PANDEMIC DYNAMICS

In this section, we describe the phenomenological procedure to estimate the evolution of the infected population.

We start with the dataset $R_{j}$ for the recovered population at day j, with j=1,…,N measuring elapsed days, with a final day N. The information of this dataset is equivalent to the cumulative integral or the sum of infected cases. The data are obtained from Ref. 24. By using the information in $R_{j}$, we can infer the infected population data as

$$I_{j}=R_{j}-R_{j-1},$$ (1)

at day j. For this work, we have used data of Ref. 24 until June 19, 2020.

For such a given dataset for the recovered population, a global dynamical behavior can be found by fitting the curve $R_{j}\to R(t)$, where now the continuous recovered population function is given by

$$R(t)=a\left(\tanh\left(b\;t+c\right)-\tanh c\right)+R_{0},$$ (2)

where a, b, and c are global constant adjustment parameters, and we have assumed that the relevant data to perform any analysis start with $R_{0}\neq 0$ by properly setting the initial time t=0 of our analysis. By global, we mean that the adjustment is for the total lapsed time N. Notice that the recovered population curve described by Eq. (2) is not equal to the approximated solution emerging from the SIR system. We show below that Eq. (2) is good global fitting for the recovered population. On the other hand, the global infected population dynamics is assumed to be found as $I_{j}\to I(t)=\dot{R}(t)$, which gives

$$I(t)=a\;b\;{\text{sech}}^{2}\left(b\;t+c\right).$$ (3)

Now, let us perform the meta-analysis of fitting Eq. (2) for the recovered population. We promote the three parameters used in Eq. (2) to meta-parameters $a\to a_{1}(t)$, $b\to a_{2}(t)$, and $c\to a_{3}(t)$. These meta-parameters are no longer global constants. Their dynamics must be obtained considering the new information that brings any new day that is added to the dataset of the recovered population. For each time j (j=1,…,N), the meta-parameters $a_{i}$ (i=1,2,3) are found by fitting the curve (2) to the data by using them as constant adjustment parameters for such time. As the amount of data grows with time, the value of each meta-parameter varies, taking into account the different behaviors that the governments or the society may have at different times. After several iterations are performed for different times and fitting curves described by Eq. (2), a regular and dynamical behavior of each meta-parameter starts to emerge. This regularity starts at some time τ for the three meta-parameters, and it depends on each particular studied case. All of this implies that meta-parameters are not globally constant in time, and now their global time-dependence $a_{i}=a_{i}(t)$ is apparent; importantly, it can be deduced.

Once this stage is reached, the complete dynamical solution for each meta-parameter is established, and solution (2) for the recovered population can now be promoted to the function

$$R_{M}(t)=a_{1}(t)\left[\tanh\left(a_{2}(t)\;t-a_{3}(t)\right)-\tanh\left(a_{3}(t)\right)\right]-R_{0},$$ (4)

which produces a dramatic departure from the solution of Eq. (2) itself.

With all the above, the new meta-parameter fitting function (4) contains more precise information on the daily changes of the recovered population compared with the fitting function (2). In other words, its derivative represents a more accurate description of the infected population curve, which can be calculated as

$$I_{M}(t)={\dot{R}}_{M}(t),$$ (5)

which anew turns out to be different from function (3).

It is shown below that when meta-parameters have achieved a regular dynamics, they all can be described in the form (i=1,2,3)

$$a_{i}(t)\approx \sum_{k=0}^{Z}a_{ki}t^{k},$$ (6)

with constant coefficients $a_{ki}$ and Z>0. Several meta-analysis produces a very good agreement with the data for Z=2. On the other hand, and remarkably, to describe the sub-epidemic behavior of different countries, it is enough to consider Z=4. This shows that any change in the information of the data evolves in an ordered way and it can be recovered through the study of the meta-parameters.

In order to quantify how both infected population fittings differ from each other, we define the global adjustment function $\epsilon =E_{M}/E_{C}$, as the ratio between $E_{M}={\sum_{j=2}^{N}\left[I_{M}(j)-I_{j}\right]}^{2}$, which is the error function for the meta-analysis of the infected population described by Eq. (5), and $E_{C}={\sum_{j=2}^{N}\left[I(j)-I_{j}\right]}^{2}$, which is the error function for the fitting of the infected population described by Eq. (3) with constant adjustment parameters. The case of ϵ<1 implies a better fitting curve for the infected population dynamics due to the meta-analysis.

## IV. APPLICATIONS OF THE METHOD

In this section, we present examples for different countries that explicitly show the strength of this phenomenological dynamical analysis and its better fitting to the existent data. It is the goal of this work to search for the explicit form of each meta-parameter for the studied countries, finding in this way the underlying dynamical structure of their pandemic scenarios, and thus determine $R_{M}$ and $I_{M}$. Thus, we show that this phenomenological model can also take into consideration the sub-epidemics occurring during the whole time lapsed for the pandemic in some countries. The sub-epidemic behavior is coded in the time dependence of meta-parameters.

With all this in mind, let us discuss the pandemic data for cumulative infectious cases as evolving over time for 11 different countries, and how the phenomenological dynamical procedure applies to each of them. We use the case of Italy to carefully explain each step in the procedure, as it is straightforwardly replicated for the other country cases. The data for every country are extracted from Ref. 24 until June 19, 2020.

### A. Italy

**FIG. 1.**
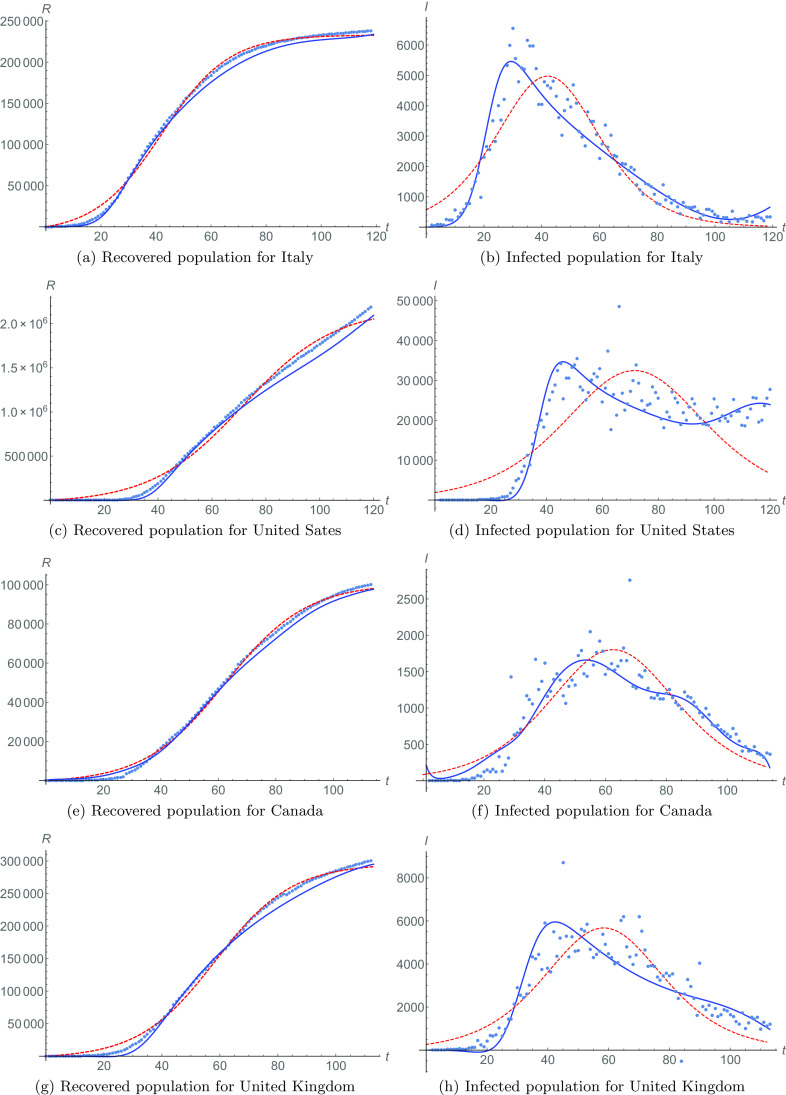
Recovered and infected populations for Italy [Figs. 1(a) and 1(b), respectively], the United Sates [Figs. 1(c) and 1(d)], Canada [Figs. 1(e) and 1(f)], and the United Kingdom [Figs. 1(g) and 1(h)]. Data are shown in dotted lines. Fittings (2) and (3) are shown in red dashed lines for recovered and infected populations, respectively. Phenomenological fittings (4) and (5), with meta-parameters, are shown in blue solid lines for recovered and infected populations, respectively.

The data for the recovered population in Italy are shown by the dotted line in Fig. 1(a), with N=119. The red dashed line shows the fitting of function (2), with parameters $R_{0}=17$, a=120072, b=0.04143, and c=−1.74422.

**FIG. 2.**
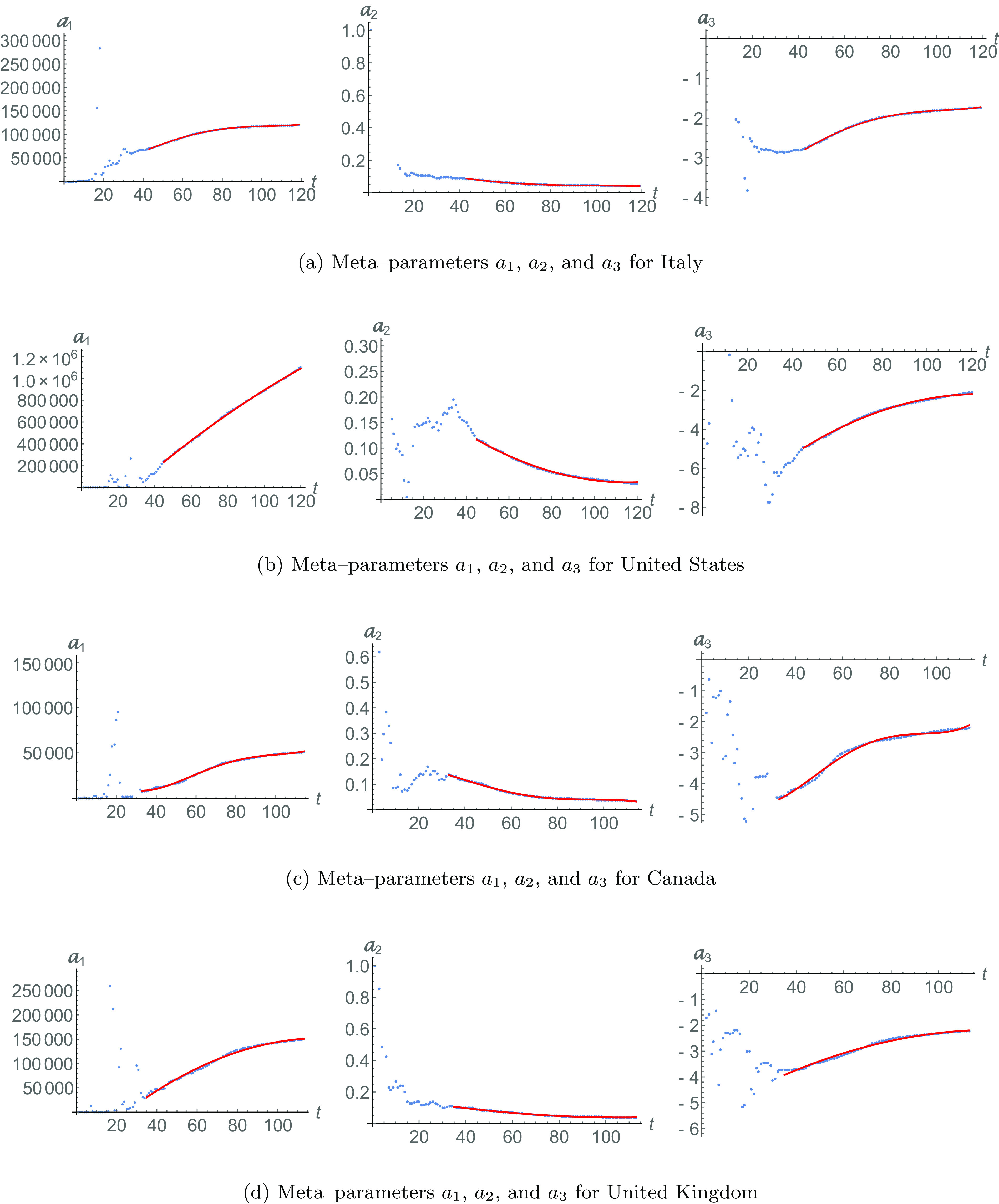
Meta-parameters for Italy [Fig. 2(a)], the United States [Fig. 2(b)], Canada [Fig. 2(c)], and the United Kingdom [Fig. 2(d)]. Data are shown in dotted lines. The regular dynamical behavior of meta-parameters (starting in time τ) is shown in red solid lines. They evolve following the quadratic form (6).

Consider now the blue solid line in Fig. 1(a). It describes the meta-analysis fitting of Eq. (4) for the recovered population since the day τ=43. This is the day in which the meta-parameters start to behave regularly, as it can be seen in Fig. 2(a). The meta-parameters are calculated for each day (from t=0 for $R_{0}$), taking into account all previous days. Thus, each new calculated meta-parameter contains the information of any previous change. Before day τ, there is no regular pattern in the evolution of meta-parameters. However, after day τ, a very distinctive regular dynamical behavior emerges. For the current case, after day τ=43, the meta-parameters’, $a_{1}(t)$, $a_{2}(t)$, and $a_{3}(t)$, time-dependence behavior of Eq. (6) is obtained for Z=4, shown by red lines in Fig. 2(a). As an example for this case, we display the coefficients of the form (6). These are $a_{01}=15\;076$, $a_{11}=-21.2629$, $a_{21}=54.6027$, $a_{31}=0.689\;66$, $a_{41}=0.002\;48$, $a_{02}=0.218\;57$, $a_{12}=-0.004\;26$, $a_{22}=0.000\;028$, $a_{32}=2.975\;37$, $a_{42}=-5.8174\times 10^{-10}$, $a_{03}=-4.259\;59$, $a_{13}=0.022\;51$, $a_{23}=0.000\;63$, $a_{33}=-9.8595\times 10^{-6}$, and $a_{43}=3.7844\times 10^{-8}$. These meta-parameters are used in Eq. (4) to obtain the solid blue fit in Fig. 1(a).

Notice that the fitting precision for the slope of the recovered population becomes more accurate as it grows; i.e., a better fit for its derivative is achieved compared to the fitting of Eq. (2). This implies that it contains better information on the number of infected individuals. This is shown in Fig. 1(b), where the data represented by dots are the infected population calculated according to Eq. (1). The red dashed line is the infected population fit (3), with their respective parameters. Notice also that this fit is just proportional to the approximated solution for the infected population in the SIR model. On the other hand, the solid blue line in Fig. 1(b) corresponds to the meta-analysis fitting of Eq. (5) for the infected population, with meta-parameters described by the above coefficients. This fitting reproduces better the global behavior of the evolution of the infected population in Italy, just by considering the last part of the growing on recovered cases (for times t>τ).

The evaluation of the global adjustment function gives ϵ=0.39021, allowing us to establish that the meta-parameter fitting for the infected population is superior to fit (3).

### B. United States

The recovered population for the United States is shown in Fig. 1(c). The data with N=120 are represented by a dotted line, while the red dashed line shows the fitting (2), with parameters $R_{0}=16$, a=243386, b=0.11813, and c=−4.93365. The blue solid line is the fitting (4) since τ=45, when the meta-parameters start to have a regular evolution (6) with Z=2, as it can be seen in Fig. 2(b) in red lines.^25^

In this case, something similar to the previous case occurs. The fitting of Eq. (4), with their respective meta-parameters, is not much better than the fit of Eq. (2) for the recovered population. However, its slope is in much better agreement with the growth rate for the recovered data. This implies that our meta-analysis gives a better fit for the infected population compared to the extracted data from Eq. (1). This can be seen in Fig. 1(d). In this case, the red dashed line represents the fitting (3) for the infected population, while the blue solid line is our meta-analysis fitting (5) using meta-parameters (6).

The fit due to meta-parameters is so dramatic that when the global solution (3) shows a decrease on the infected population, the meta-analysis shows that the rate is not slowed down, but it is increasing. For times t⪆80, our meta-analysis has been able to extract the information of the sub-epidemics occurring during the complete time scale of the pandemic. Sub-epidemics are usually modeled as overlapping of several epidemic episodes. These are described as multimodal epidemic events satisfying coupled differential equation models.^19^ However, in our meta-analysis model, a large sub-epidemic behavior emerges from the study of the evolution of meta-parameters, without invoking any specific model designed to describe these kinds of epidemics. This is a strength of our model, as no *a priori* knowledge of the evolution of the pandemic is needed in order to discover the sub-epidemic events in a society. In the case of the United States, this sub-epidemic event is happening right now.

Furthermore, the meta-analysis fitting is better than model (3), as the global adjustment function is ϵ=0.27888.

### C. Canada

The recovered population data for Canada are shown with the dotted line in Fig. 1(e). The red dashed line is the fit of Eq. (2) with N=114 and parameters $R_{0}=12$, a=−50965.9, b=0.03536, and c=−2.20783.

Anew, the blue solid line is our meta-analysis fitting of Eq. (4), using the meta-parameters that are described by a regular dynamics (6), with Z=4 and starting in τ=33, as red lines in Fig. 2(c). The blue solid line for recovered cases indicates a better approximation to the growing (slope) of such data.

The infected population is depicted in Fig. 1(f), where the data are obtained from Eq. (1), while in the red dashed line is the fitting (3), and in the blue solid line, we have the fitting of Eq. (5) with the meta-parameters. Once again, the meta-analysis shows several sub-epidemic events that cannot be obtained by fitting (3). This sub-epidemics appears only when the time-dependent evolution of the meta-parameter of Fig. 2(c) is found.

Finally, with ϵ=0.81282 for this case, the meta-analysis represents a better fit for the global evolution of the infected population.

### D. United Kingdom

The recovered population data are shown in Fig. 1(g), while the infected population data are shown in Fig. 1(h), both of them in dotted lines. The recovered population data fit (2), in a red line in Fig. 1(g), is achieved with N=113 and parameters $R_{0}=16$, a=149676, b=0.03791, and c=−2.21432.

The blue solid line in Fig. 1(g) represents the fit of meta-analysis (4) for meta-parameters (6) that have achieved regular evolution for τ=35 and Z=2 [see Fig. 2(d)]. The information in the meta-analysis is used for the infected population in comparison with data (1). In Fig. 1(h), the solid blue line for meta-parameter fitting (5) shows a better correspondence than the fit given by Eq. (3) with constant parameters. This is confirmed by evaluating ϵ=0.84056.

### E. Spain

**FIG. 3.**
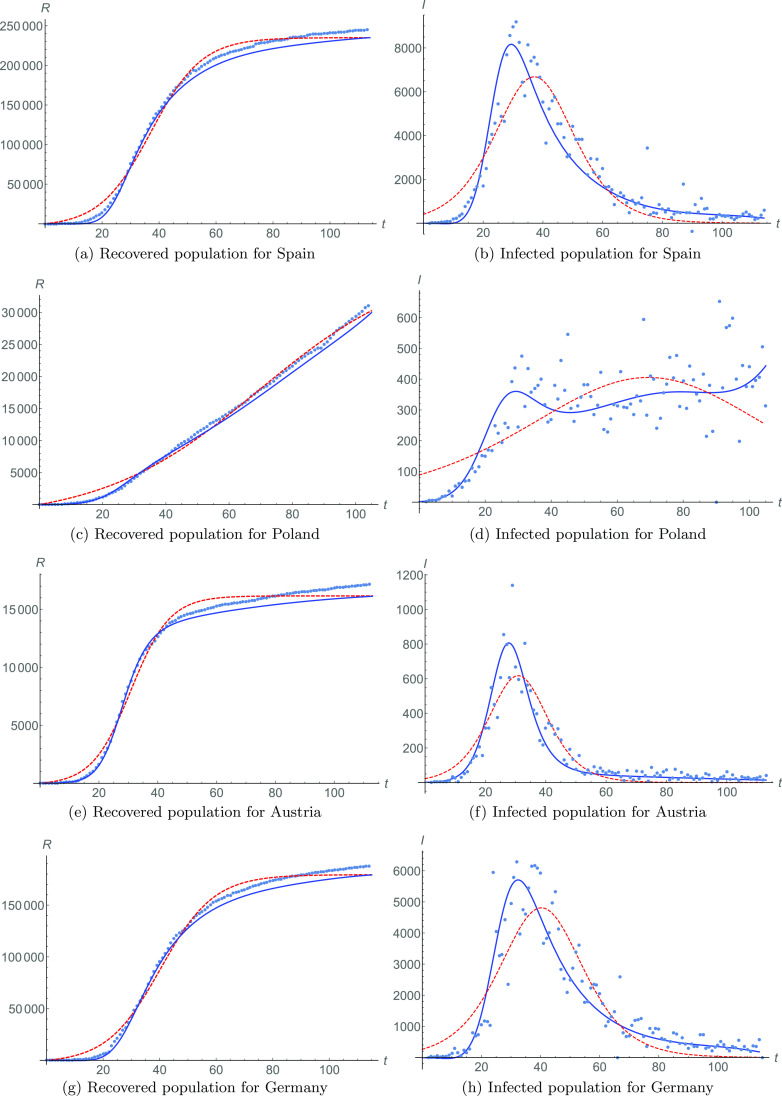
Recovered and infected populations for Spain [Figs. 3(a) and 3(b), respectively], Poland [Figs. 3(c) and 3(d)], Austria [Figs. 3(e) and 3(f)], and Germany [Figs. 3(g) and 3(h)]. Data are shown in dotted lines. Fittings (2) and (3) are shown in red dashed lines for recovered and infected populations, respectively. Phenomenological fittings (4) and (5), with meta-parameters, are shown in blue solid lines for recovered and infected populations, respectively.

**FIG. 4.**
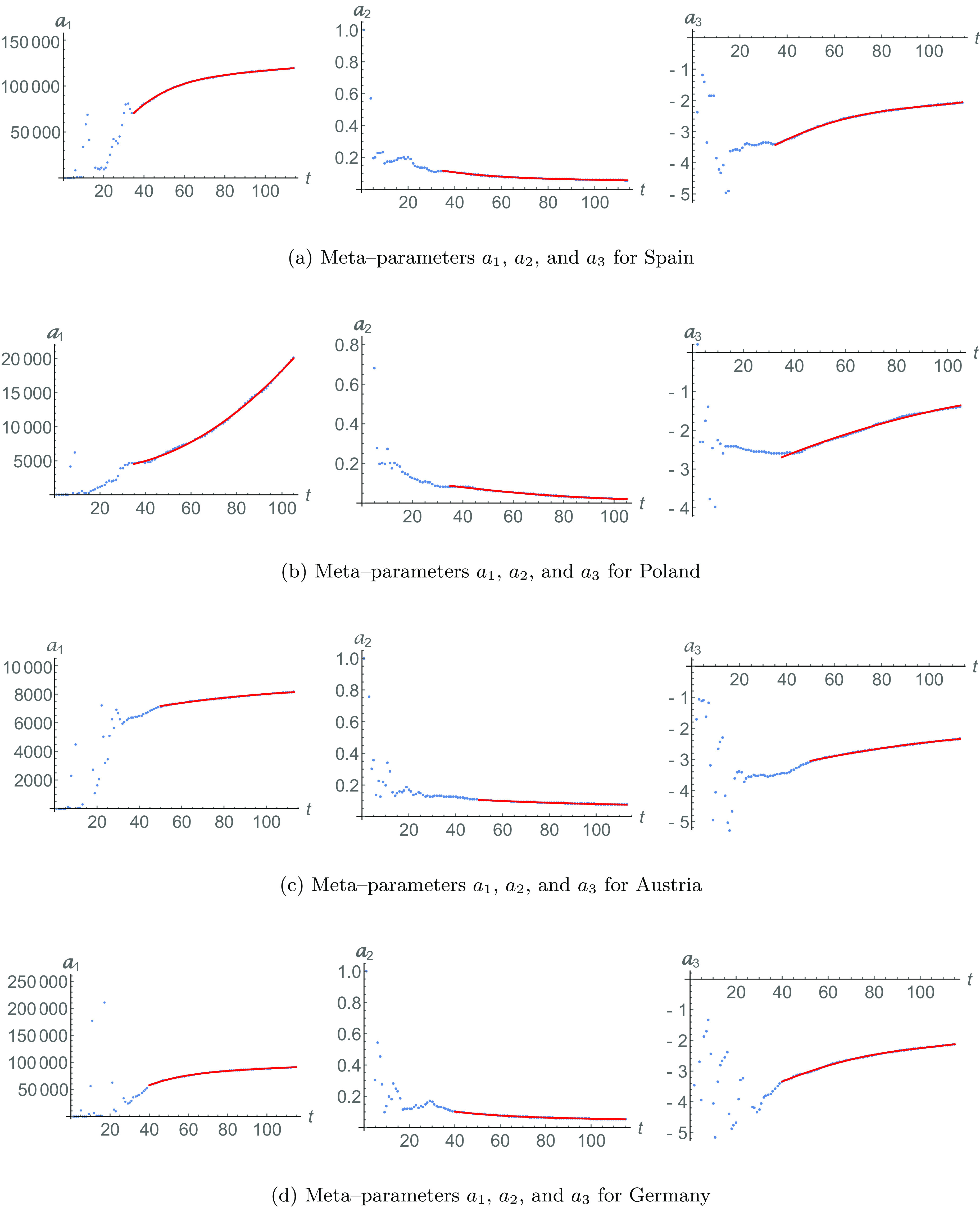
Meta-parameters for Spain [Fig. 4(a)], Poland [Fig. 4(b)], Austria [Fig. 4(c)], and Germany [Fig. 4(d)]. Data are shown in dotted lines. The regular dynamical behavior of each meta-parameter (starting in time τ) is shown in red solid lines. They evolve following the quadratic form (6).

In Fig. 3(a), we show the recovered population data (in a dotted line). The red dashed line is the fitting (2) for the recovered population with N=114 and parameters $R_{0}=9$, a=119352, b=0.05597, and c=−2.07708. Similarly, the blue solid line is fitting (4) with meta-parameters (6) with Z=4 and starting in τ=35. We can see in Fig. 4(a) that the meta-parameters are described by quartic functions.

Using this, we can calculate the behavior of the number of infected individuals, shown in Fig. 3(b). The infected population data (dotted line) are given by Eq. (1), while fit (3) is in a red dashed line and meta-analysis fit (5) is in a solid blue line. We obtain that ϵ=0.49292, showing that the fit based in meta-parameters is again superior to the one based in time-independent parameters.

### F. Poland

The data for the recovered population of Poland are plotted in Fig. 3(c) in a dotted line. Also, the red dashed line fits Eq. (2) for the recovered population with N=105 and parameters $R_{0}=5$, a=20169.1, b=0.02011, and c=−1.39525. The meta-analysis fitting (4) is in a blue line with the meta-parameters (6) from Fig. 4(b), starting in τ=35, with Z=2.

This case is interesting as the slope of meta-analysis fit (4) clearly shows that the recovered population is increasing, as opposed to what can be deduced from fit (2). Similarly, the infected population data, in Fig. 3(d), show a better agreement with the fit (5) due to meta-parameters. While the fit (3) indicates a strong decrease in the infected population, the meta-analysis shows that the infected population is increasing due to sub-epidemic events. A better fit of the meta-analysis is also corroborated by ϵ=0.65004.

### G. Austria

Figure 3(e) shows the data for the recovered population in Austria. The fit (2) for N=113 and parameters $R_{0}=5$, a=8152.2, b=0.07569, and c=−2.33693 is represented by a red line. Similarly, the fit (4), represented by a blue solid line, requires the meta-parameters (6) shown in Fig. 4(c), with coefficients starting from τ=50, with Z=2.

The meta-analysis fit indicates a better adjustment for the infected population, as it is seen in Fig. 3(f). In this figure, the red dashed line is the fit (3), and the blue solid line is the fit (5). Notice that there is a better adjustment of the meta-analysis for the tail of the pandemic data for t>60. The global dynamical behavior of the infected population is better achieved by the meta-analysis, with ϵ=0.51678.

### H. Germany

The data for this country are shown in Figs. 3(g) and 3(h) for the data (dotted lines) of recovered and infected populations, respectively. Red dashed lines represent the fitting of (2) and (3) for both cases, with N=115 and $R_{0}=17$, a=90954.5, b=0.05287, and c=−2.12998. Blue solid lines represent the meta-parameter fittings of recovered (4) and infected (5) populations, with respect to the data (1). The meta-parameters (6) start from ζ=40, with Z=4.

Once more, the fit (5) is better for the infected population, as ϵ=0.59191.

### I. Portugal

**FIG. 5.**
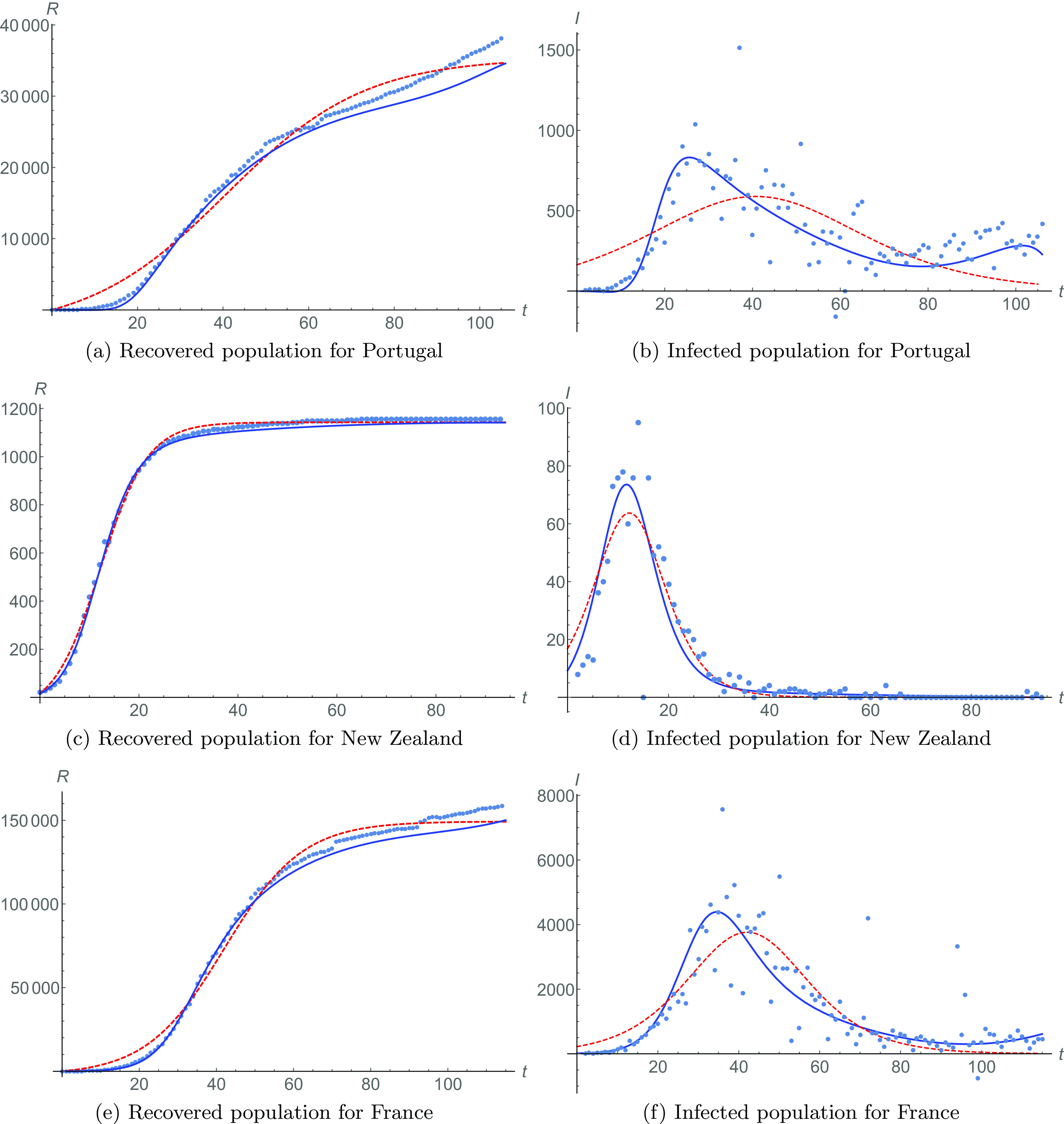
Recovered and infected populations for Portugal [Figs. 5(a) and 5(b), respectively], New Zealand [Figs. 5(c) and 5(d)], and France [Figs. 5(e) and 5(f)]. Data are shown in dotted lines. Fittings (2) and (3) are shown in red dashed lines for recovered and infected populations, respectively. Phenomenological fittings (4) and (5), with meta-parameters, are shown in blue solid lines for recovered and infected populations, respectively.

**FIG. 6.**
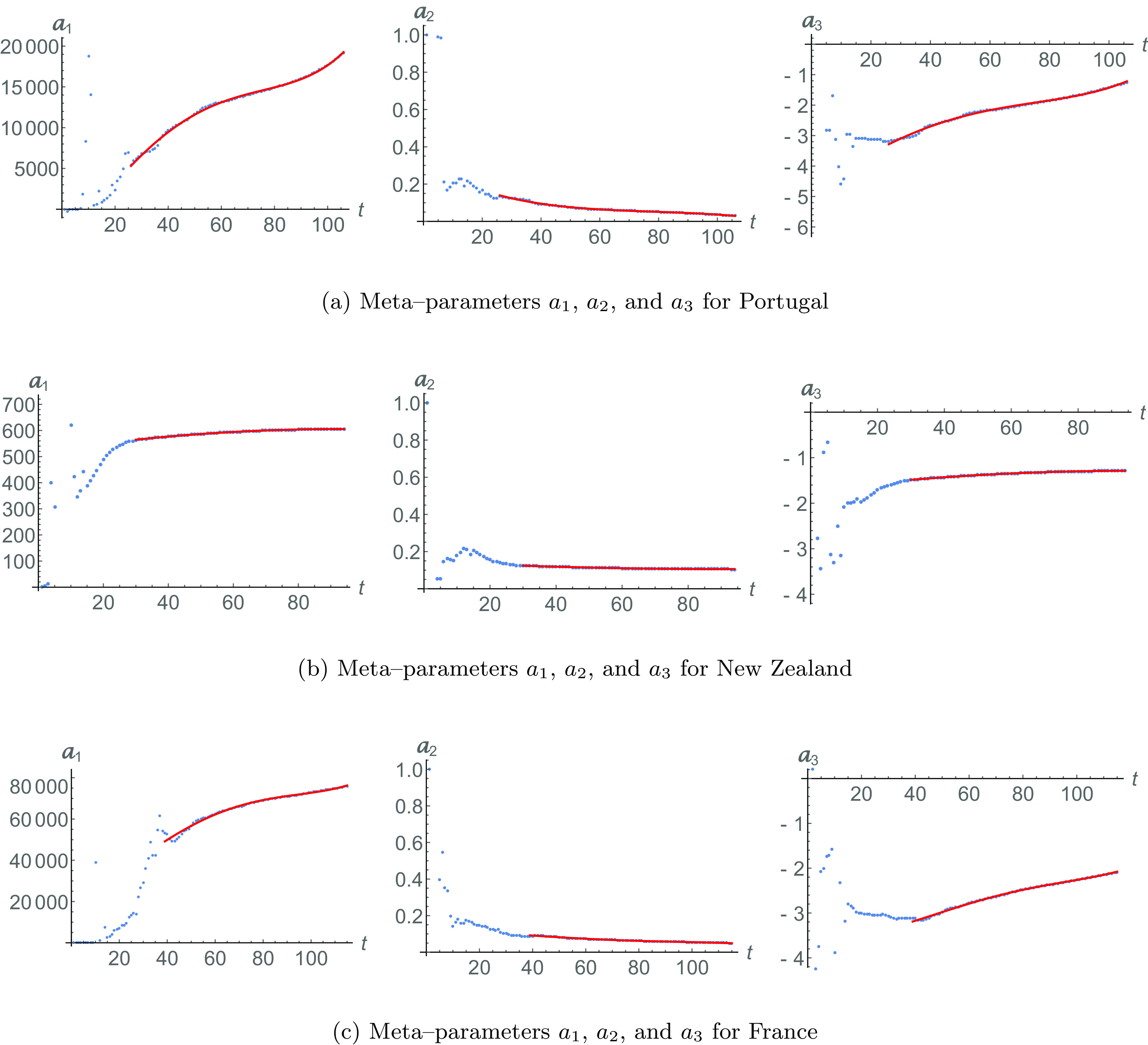
Meta-parameters for Portugal [Fig. 6(a)], New Zealand [Fig. 6(b)], and France [Fig. 6(c)]. Data are shown in dotted lines. The regular dynamical behavior of each meta-parameter (starting in time τ) is shown in red solid lines. They evolve following the quadratic form (6).

Figure 5(a) shows the recovered cases in dotted lines and the fitting (2) in red dashed lines with N=106 and $R_{0}=9$, a=19109.7, b=0.03079, and c=−1.25453. A blue solid line is fit (4), with the metaparameter shown in Fig. 6(a), with τ=26 and Z=4. Similarly, we show the different fitting (3) and (5) for infected populations in Fig. 5(b). The meta-analysis produces a better fit, as ϵ=0.50303.

Notice that, again, sub-epidemic events are present, and they are apparent only through the known evolution of meta-parameters.

### J. New Zealand

In Figs. 5(c) and 5(d), we show the recovered and infected population for New Zealand. The data are represented by dotted lines, while fittings (2) and (3) are represented by red dashed lines with N=94 and $R_{0}=20$, a=606.56, b=0.10504, and c=−1.28409. The blue solid line is the meta-analysis fitting (4) and (5), for meta-parameters of Fig. 6(b), with Z=2 and τ=30.

This case is very interesting as it is the only one in which the meta-analysis and the fitting with constant parameters almost coincide (although the meta-analysis is better with ϵ=0.88445). This is an indication of the very good health policies adopted by the government to deal with the pandemic and of the responsible behavior of the society. In this (almost ideal) case, the evolution of the infection variables behaves as a very precise mathematical model.

### K. France

The recovered and infected population data of the last country we consider, France, are shown in Figs. 5(e) and 5(f). Fittings (2) and (3) are in red dashed lines with N=115 and $R_{0}=14$, a=75771.6, b=0.04966, and c=−2.09013. The data are represented by dotted lines.

Blue lines are fittings (4) and (5) with meta-parameters from Fig. 6(c), with Z=4 and τ=39. Our meta-analysis adjustment gives ϵ=0.77846, and it predicts a slight increase of the infected population.

## V. DISCUSSION

We have presented a new approach to deal with infection propagation data by allowing the parameters to become time-dependent. We are not able to produce a dynamical model for the time evolution of parameters, mainly due to intrinsic difficulty associated with unforeseeable government policies and population behavior. Instead, we have been able to produce a method that may be successfully applied to current data. These data have been informed by 11 different countries that have implemented different mitigation policies to fight the COVID-19 infection with different population reactions.

All of the cases of infected populations studied above exhibit the same feature. The meta-analysis shows effectively the capture of the daily variations of cases. In other words, we have shown that using meta-parameters, we can *integrate* the recovered population without using any pre-existing model. Our proposal produces global results, as soon as the regular behavior of meta-parameters is found. Thus, the meta-analysis works for all data and not only for an arbitrary particular range in the evolution of recovered cases, for example, when the growing of recovered cases behaves as a power-law.^26^ This proposed model can also detect sub-epidemic events occurring during the pandemic, which is done without the assumption of any model based on differential equations.

Our model has two main key characteristics that need to be considered to produce sensible results. The first one is the starting model required to fit the data. In our case, we use a tanh fit (2), but other more complex models, as modified logistic ones, can be used as a starting point for the meta-analysis. This can improve the fitting done by the meta-parameters. The second one is the high sensitivity of our model to well-established data for cases. The infection data that are informed in countries where the governments have not been able to handle the pandemic or have introduced sudden infection-related policy changes, or where the population has not abided by the government set containment rules, are difficult to describe by the approach presented here (or almost any other method). This is because no regularity can be found when the meta-parameters are analyzed. This is the case of, for example, Chile, where the health authority’s policies designed to address the pandemic and the response of the society have proved unsuccessful up to now.

## Data Availability

The data that support the findings of this study are openly available in Our World in Data at ourworldindata.org, Ref. 24.
